# Health-related quality of life in French adolescents and adults: norms for the DUKE Health Profile

**DOI:** 10.1186/1471-2458-11-401

**Published:** 2011-05-27

**Authors:** Cédric Baumann, Marie-Line Erpelding, Christine Perret-Guillaume, Arnaud Gautier, Stéphanie Régat, Jean-François Collin, Francis Guillemin, Serge Briançon

**Affiliations:** 1 Nancy-Université, Université Paul Verlaine Metz, Université Paris Descartes, EA 4360 Apemac, Nancy, France; 2 CHU Nancy, Hôpitaux de Brabois, Epidémiologie et Evaluation Cliniques, Vandœuvre-les-Nancy, France; 3 CHU Nancy, Hôpitaux de Brabois, service de gériatrie, Vandoeuvre-les-Nancy, France; 4 INPES, département Observation et Analyse des Comportements de Santé, Paris, France

**Keywords:** Health-related quality of life, Duke Health Profile, norms, adolescent, adult, French population

## Abstract

**Background:**

The continual monitoring of population health-related quality of life (HRQoL) with validated instruments helps public health agencies assess, protect, and promote population health. This study aimed to determine norms for the French adolescent and adult general population for the Duke Health Profile (DUKE) questionnaire in a large representative community sample.

**Methods:**

We randomly selected 17,733 French people aged 12 to 75 years old in 2 steps, by households and individuals, from the National Health Barometer 2005, a periodic population study by the French National Institute for Prevention and Health Education. Quality of life and other data were collected by computer-assisted telephone interview.

**Results:**

Normative data for the French population were analyzed by age, gender and self-reported chronic disease. Globally, function scores (best HRQoL=100) for physical, mental, social, and general health, as well as perceived health and self-esteem, were 72.3 (SEM 0.2), 74.6 (0.2), 66.8 (0.1), 71.3 (0.1), 71.3 (0.3), 76.5 (0.1), respectively. Dysfunction scores (worst HRQoL=100) for anxiety, depression, pain and disability domains were 30.9 (0.1), 27.6 (0.2), 34.3 (0.3), 3.1 (0.1), respectively.

**Conclusion:**

The French norms for adolescents and adults for the DUKE could be used as a reference for other studies assessing HRQoL, for specific illnesses, in France and for international comparisons.

## Background

Quality of life (QoL) is defined by the World Health Organization (WHO) as ''the perception that an individual has of his or her place in life, within the context of the culture and system values in which he or she lives, and in relation to the objectives, expectations, standards and concerns of this individual'' [1]. Health-related quality of life' (HRQoL) [2,3] can be defined ''as an integrative measure of physical and emotional well-being, level of independence, social relationships and their relationship to salient features of their environment'' [1]. The conceptualization of HRQoL is both objective and subjective, so its measurement requires reference to varied and complex areas, depending on the perspective. For example, social workers will assess QoL from a different perspective than medical workers. HRQoL measurement can also be very personal because experiences, beliefs, and expectations and perceptions influence how individuals think and behave [4].

HRQoL is a multidimensional concept that relates specifically to a person's health, to the measure of its functioning, well-being and general health perception in physical, psychological, and social domains [2]. HRQoL measures are used to determine the burden of disease in economic analyses [5,6] and have become an important target in medical care for assessing treatment outcomes in chronic disease and an important outcome criterion in randomised clinical trials, especially oncology [1]. In addition, HRQoL instruments can be used in medical practice to improve the physician-patient relationship, in health services evaluation, in research and in policy making.

Many HRQOL instruments, both generic and specific for various illnesses, have been developed to survey the various domains of life that ill health can affect [7].

Most generic instruments are for adults, such as the WHOQOL [8], the Sickness Impact Profile [9], the Nottingham Health Profile [10], the SF-36 [11], and the Duke Health Profile (DUKE) [12-14]. However, whether such generic instruments are suitable for young French people is unknown. To compare the adolescent and adult quality of life, the French Committee for Health Promotion, in 1998, 2000 and in 2005 [15], used a version of the DUKE suited to assess quality of life in the 12-19 age group.

The DUKE is a cross-culturally adapted, valid and useful measure of perceived health in adolescents and adults [12]. One of the obstacles to the success of large surveys is the extensive time needed to complete them (by phone conversations or self-administered). The DUKE is a 17-item short questionnaire, self-administered or interviewer-administered, developed and validated in primary care to measure patient-reported HRQoL, or functional health status, during 1 week [13,14] and may be more suitable than the SF-36 for older inpatients [16-18]. Its feasibility and acceptability were reported to be good for patients with dementia [19]. As well, another study found the DUKE significantly better accepted than the SF-36 by young patients [20]. Finally, the DUKE allows for briefly exploring dimensions of self-perceived health such as self-esteem, anxiety and depression not proposed by other tools [21].

There is an interest in finding a simple short, self-reporting measure of HRQoL in healthy adolescents that is in the French language. The DUKE score has been used primarily for research in the clinical setting, both as a predictor of health-related outcomes and as an outcome [22-24]. The original DUKE was developed in English (United States) and was validated primarily in the United States. Subsequently, the DUKE has been translated into 17 other languages and language variations such as Afrikaans, Chinese, Dutch, Dutch (Belgium), English (UK), French, French (Canada), German, Italian, Korea, Norwegian, Polish, Portuguese, Spanish, Swedish, Thai and Vietnamese. It has been translated in French and used extensively by the Public Health School of Nancy (France) [12,25-29].

Medical and scientific committees need validated instruments to assess HRQoL, but general population norms are lacking, which limits their full use in research and clinical practice. Community norms of HRQoL are important because they provide a base level of HRQoL to compare illness groups or individuals' HRQoL to expected values. To our knowledge, norms for the DUKE for all countries are lacking.

We aimed to use the DUKE to determine HRQoL norms for French adolescents and adults and analyze these by gender, age and self-reported chronic disease.

## Methods

### Data source

Since 1992, the French National Health Barometer, a 5-year periodic study by the French National Institute for Prevention and Health Education (INPES), has surveyed behaviours, attitudes, opinions and knowledge about health (e.g., alcohol consumption, tobacco use, drug consumption, physical activity) and evolution of the health of adolescents and adults in France. The whole questionnaire includes more than 400 questions. Data for the 2005 National Health Barometer were collected between October 14, 2005 and February 12, 2005. This survey was carried out in France by use of a computer-assisted telephone interview (CATI) system with a sample of 30,514 people aged 12 to 75 years who spoke French. Households received a letter in advance to explain the purpose of the survey and to encourage people in the household to take part. The eligible subject within each household whose next birthday was nearest the interview day was selected to answer the questions [30]. All data collected were anonymous and self-reported. Subjects were asked to isolate themselves before the interview began. The mean duration of an interview was about 40 minutes for landline phones.

Young people (younger than 15 years) had to be accompanied by their mother or father to participate. Parents were asked to consent to their child's participation and that the child could be isolated to speak more freely.

The INPES commissioned the "EA4360 Apemac", a French research team specialised in HRQoL studies (School of Public Health, Nancy, France), to analyse the data and determine norms [15].

This population-based survey was approved by the French National Institutional Review Board (Commission Nationale Informatique et Liberté).

### Sampling

Of 30,514 participants in the 2005 Health Barometer survey, 26,672 were contacted by landline phone to answer all questions of the Health Barometer, and 3,842 persons, without a landline phone, were contacted by their mobile phone to answer questions related only to tobacco, alcohol and illegal drug use because by the year 2000, more people had only a mobile phone. When the Barometer started, questioning all participants by mobile phone for more than 20 minutes was difficult (problems with the battery, attention, satellite range). So, the researchers decided to ask questions about sociodemographic characteristics and tobacco, alcohol, and illegal drug consumption only to limit the duration of the interview.

**Figure 1:**
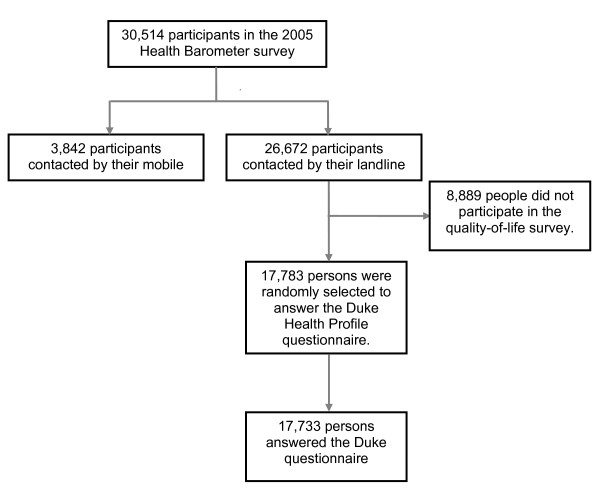
**Selection of the participants in the health-related quality-of-life survey**.

Among the 26,672 participants contacted by landline phone, 17,783 (two-thirds of the sample) were randomly selected to participate in the QoL survey by the DUKE. Among these, 17,733 responded to the DUKE. The 8,889 participants not randomized responded to another HRQoL questionnaire (WHOQOL-brief) (see figure 1).

### Duke Health Profile questionnaire

HRQoL was assessed by use of a French validated version of the DUKE (Table 1), a 17-item generic self-reporting instrument, with question responses according to a 3-point Likert scale, which covers a 1-week time frame [12]. The DUKE includes 10 domains. Six domains are about health function: physical health (items 8-12), mental health (items 1, 4, 5, 13, 14), social health (items 2, 6, 7, 15, 16), general health (aggregation of physical, mental and social health measures to indicate overall well-being) (15 items), perceived health (item 3) and self-esteem (items 1, 2, 4, 6, 7), with high scores indicating better HRQoL; and 4 are about health dysfunction: anxiety (items 2, 5, 7, 10, 12, 14), depression (items 4, 5, 10, 12, 13), pain (item 11) and disability (item 17), with high scores indicating greater dysfunction. The DUKE is suitable for computerised telephone administration by a trained interviewer. It can be completed in a short time and has good acceptability [14,31].

**Table 1:** Content of the Duke Health Profile questionnaire

**Item **(*French version)*	Dimension *
1. **I like who I am** *(Je me trouve bien comme je suis)*	Mental health, self-esteem, anxiety
2. **I am not an easy person to get along with** *(Je ne suis pas quelqu'un de facile à vivre)*	Social health
3. **I am basically a healthy person** *(Au fond, je suis bien portant)*	Perceived health
4. **I give up too easily** *(Je me décourage trop facilement)*	Mental health, self-esteem, depression
5. **I have difficulty concentrating** *(J'ai du mal à me concentrer)*	Mental health, anxiety, depression
6. **I am happy with my family relationships** *(Je suis content de ma vie de famille)*	Social health, self-esteem
7. **I am comfortable being around people** *(Je suis à l'aise avec les autres)*	Social health, anxiety
8. **Would you have any physical trouble or difficulty : Walking up a flight of stairs** *(Vous auriez du mal à monter un étage)*	Physical health
9. **Would you have any physical trouble or difficulty : Running the length of a football field** *(Vous auriez du mal à courir une centaine de mètres)*	Physical health
10.**How much trouble have you had with: sleeping**. *(Vous avez eu des problèmes de sommeil)*	Physical health, anxiety, depression
11. **How much trouble have you had with: hurting or aching in any part of your body** *(Vous avez eu des douleurs quelque part)*	Physical health, pain
12. **How much trouble have you had with: getting tired easily** *(Vous avez eu l'impression d'être vite fatigué(e))*	Physical health, anxiety, depression
13. **How much trouble have you had with: feeling depressed or sad** *(Vous avez été triste ou déprimé(e))*	Mental health, depression
14. **How much trouble have you had with: nervousness** *(Vous avez été tendu(e) ou nerveux(se))*	Mental health, anxiety
15. **How often did you: socialize with other people (talk or visit with friends or relatives)**. Vous vous êtes retrouvé(e) avec les gens de votre famille qui n'habitent pas chez vous, ou avec des copains en dehors de l'école (posée aux 12-17 ans) Vous avez rencontré des parents ou des amis au cours de conversations ou de visites (posée aux 18 ans et plus)	Social health
16. **How often did you: take part in social, religious, or recreation activities (meetings, church, movies, sports, parties)**. *(Vous avez eu des activités de groupes ou de loisirs)*	Social health
17. **How often did you: stay in your home, a nursing home, or hospital because of sickness, injury, or other health problem** *(Vous avez dû rester chez vous ou faire un séjour en clinique ou à l'hôpital pour raison santé)*	Disability

* The general health dimension consists of all items, except items 3 and 17.

### Other data collected

Like many other authors [7,32], we considered age, gender and self-reported chronic disease to determine norms.

### Statistical analysis

Questionnaires were coded and calculated according to instructions in the DUKE manual [14]. The score for each dimension is the sum of the scores for the items, standardized from 0 to 100. For the 6 health dimension scores, 100 indicates the best HRQoL, whereas for the 4 dysfunction dimension scores, 100 indicates the greatest dysfunction. Missing dimension scores were imputed if scores were missing for < 50% of items for a dimension, using the mean score of the items completed within that dimension. Scores were analysed for the whole sample and then after stratification by gender, age and self-reported chronic disease.

Norms for the DUKE for French adolescents and adults are presented as means, standard deviation (SD), standard error of the mean (SEM), median (interquartile range), minimum, maximum, and percentage of floor and ceiling effect. In this study, with lack of consensus, floor and ceiling effects were considered present if more than 10% of the respondents achieved the highest or lowest score, and strong effects if more than 30% of the respondents achieved the highest or lowest score.

Qualitative variables were compared by Student's *t *test, with Bonferroni correction. Interaction of gender, age groups and self-perceived chronic disease with HRQoL was analyzed by linear regression models. Only strong interactions are presented (p < 0.01).

Data and *t *test values were weighted by the number of eligible persons in the household and by the French population structure imputed from 1999 INSEE (National Institute for Statistic and Economic surveys) National Census data. In this way, the sample was representative of the French general population between 12 and 75 years old who speak French and have a landline phone.

Internal consistency was assessed by Cronbach's α, an inter-item correlation statistic ranging from 0-1, except for perceived health, pain and disability domains, which contain only one item. Higher values indicate that items on a domain are correlated and therefore the scale measures an underlying single dimension of the questionnaire. A Cronbach α of ≥ 0.5 is usually considered acceptable [33], but Nunnally recommends values of ≥ 0.7 [34].

Statistical analysis involved use of SAS v9.1 (SAS Inc., Cary, NC).

## Results

### Description of the sample

Table 2 shows the characteristics of the observed sample and after weighting by gender, age, geographic area and size of community. The response rate to the HRQoL survey was close to 100%. Among 17,783 randomly selected people, completed questionnaires were obtained from 17,733 subjects questioned by the CATI system. Participants of the HRQoL survey (n = 17,733) and people not randomly selected (n = 9,539) did not differ in age, gender or self-reported chronic disease. After weighting by the 1999 INSEE National Census data, 49.1% of the sample were males. Adolescents (12-17 years old) represented 10.5% of the sample, young adults (18-24 years old) 11.1% and elderly people (65-75 years old) 12%. Self-reported chronic disease prevalence was 21.7%.

**Table 2:** Characteristics of the sample

	**Men**			**Women**			**Total**		
			
	**n observed**	% corrected*	**n 1999 NCD**	**n observed**	% corrected*	**n 1999 NCD**	**n observed**	% corrected*	**n 1999 NCD**
									
	7425	49,1	22 828 184	10308	50,9	23501064	17733	-	46329248
									
Age, years									
12-17	670	11,5	2,360,572	737	9,6	2256154	1407	10,5	4616726
18-24	638	11,9	2706126	810	10,4	2628525	1448	11,1	5334651
25-34	1400	17,8	4201394	1927	18,9	4215311	3327	18,4	8416705
35-44	1404	17,6	4246510	1919	19,6	4337674	3323	18,6	8584184
45-54	1320	18,1	4081008	1776	17,5	4110160	3096	17,8	8191168
55-64	1125	11,2	2684944	1730	12	2798797	2855	11,6	5483741
65-75	868	11,9	2284992	1409	12,2	2859393	2277	12,1	5144385
Chronic disease									
Yes	1619	20,6	4702606	2534	22,8	5358243	4153	21,7	10053447
No	5798	79,4	18125578	7761	77,2	18142821	13559	78,3	36275801

*weighted by number of eligible persons in the household and imputed from 1999 INSEE National Census Data (1999 NCD)

### Internal consistency

Internal consistency ranged from poor to good. The Cronbach α was 0.34 for social health, 0.46 for self-esteem, 0.57 for anxiety, 0.61 for depression, 0.62 for physical health, 0.63 for mental health, and 0.71 for general health. The Cronbach α for adolescents was lower than or equal to that for adults for dimensions.

### Description of norms by gender, age and self-reported chronic disease

The HRQoL norms globally, by gender and by age are in Table 3. In summary, mean function scores for physical, mental, social, and general health, as well as perceived health and self-esteem, were 72.3 (SEM 0.2), 74.6 (0.2), 66.8 (0.1), 71.3 (0.1), 71.3 (0.3), 76.5 (0.1), respectively. Dysfunction scores for anxiety, depression, pain and disability were 30.9 (0.1), 27.6 (0.2), 34.3 (0.3), 3.1 (0.1), respectively. Scores for men were always higher than those for women, except for social health and disability dimensions.

**Table 3:** HRQoL norms* in French general population from 12 to 75 years old (n = 17,733)

	**Men**								**Women**								**Total**
			
	**12-17**	**18-24**	**25-34**	**35-44**	**45-54**	**55-64**	**65-75**	**Total**	**12-17**	**18-24**	**25-34**	**35-44**	**45-54**	**55-64**	**65-75**	**Total**	
																
																	
																	
**Physical health**																	
n=	670	637	1396	1403	1316	1121	863	7406	733	809	1927	1910	1770	1721	1381	10251	17657
Mean	80.7	79.3	79.0	77.6	74.0	75.9	71.1	76.8	72.9	71.3	70.1	69.2	66.1	64.8	61.3	67.9	72.3
Standard deviation	19.1	20.1	17.6	18.1	22.2	18.2	23.1	19.9	20.2	20.6	18.9	20.0	20.1	18.0	19.5	19.7	20.3
Error standard of the mean	0.6	0.6	0.4	0.5	0.6	0.6	0.7	0.2	0.7	0.7	0.5	0.5	0.5	0.5	0.6	0.2	0.2
Percentile 25 th	70.0	70.0	70.0	70.0	60.0	70.0	60.0	70.0	60.0	60.0	60.0	60.0	50.0	50.0	50.0	60.0	60.0
Median	80.0	80.0	80.0	80.0	80.0	80.0	70.0	80.0	70.0	70.0	70.0	70.0	70.0	70.0	60.0	70.0	80.0
Percentile 75th	90.0	90.0	90.0	90.0	90.0	90.0	90.0	90.0	90.0	90.0	80.0	80.0	80.0	80.0	80.0	80.0	90.0
Minimum	20.0	0.0	0.0	0.0	0.0	0.0	0.0	0.0	0.0	0.0	0.0	0.0	0.0	0.0	0.0	0.0	0.0
Maximum	100.0	100.0	100.0	100.0	100.0	100.0	100.0	100.0	100.0	100.0	100.0	100.0	100.0	100.0	100.0	100.0	100.0
Floor effect (%)	0.0	0.3	0.1	0.2	0.8	0.3	1.0	0.4	0.3	0.2	0.4	0.7	0.8	0.8	1.1	0.6	0.5
Ceiling effect (%)	18.0	15.6	17.1	14.4	12.8	17.0	10.1	14.9	11.2	8.4	8.7	7.7	7.0	8.1	3.9	7.8	11.3
																	
**Mental health**																	
n=	668	637	1398	1401	1311	1123	856	7394	734	809	1924	1909	1766	1718	1387	10247	17641
Mean	74.3	75.2	80.0	79.5	78.2	81.5	81.0	78.6	65.8	67.5	72.0	71.2	70.2	72.3	74.1	70.7	74.6
Standard deviation	22.7	24.6	18.4	18.3	19.6	16.6	18.6	19.6	23.5	22.1	19.3	20.4	20.1	17.1	18.8	19.9	20.2
Error standard of the mean	0.7	0.8	0.5	0.5	0.5	0.5	0.6	0.2	0.8	0.7	0.5	0.5	0.5	0.5	0.6	0.2	0.2
Percentile 25 th	60.0	60.0	70.0	70.0	70.0	70.0	70.0	70.0	50.0	50.0	60.0	60.0	60.0	60.0	60.0	60.0	60.0
Median	80.0	80.0	80.0	80.0	80.0	90.0	80.0	80.0	70.0	70.0	80.0	70.0	70.0	80.0	80.0	70.0	80.0
Percentile 75th	90.0	90.0	90.0	90.0	90.0	100.0	100.0	90.0	80.0	80.0	90.0	90.0	90.0	90.0	90.0	90.0	90.0
Minimum	0.0	0.0	0.0	0.0	10.0	0.0	10.0	0.0	0.0	0.0	0.0	0.0	0.0	0.0	0.0	0.0	0.0
Maximum	100.0	100.0	100.0	100.0	100.0	100.0	100.0	100.0	100.0	100.0	100.0	100.0	100.0	100.0	100.0	100.0	100.0
Floor effect (%)	0.1	0.1	0.2	0.0	-0.0	0.1	0.0	0.1	0.4	0.2	0.4	0.1	0.8	0.5	0.5	0.4	0.2
Ceiling effect (%)	12.2	15.4	21.6	20.5	19.7	27.4	25.2	20.3	8.6	7.6	11.0	12.4	13.0	15.3	15.8	12.1	16.2
																	
**Social health**																	
n=	669	634	1388	1387	1296	1118	841	7333	730	807	1923	1901	1756	1699	1378	10194	17527
Mean	70.3	68.1	68.7	66.4	65.7	64.9	64.4	66.9	65.7	66.7	68.6	67.0	65.5	66.7	65.7	66.7	66.8
Standard deviation	20.2	22.8	18.2	18.1	18.9	16.7	19.2	18.9	18.6	19.5	15.9	17.1	16.7	14.6	15.9	16.6	17.6
Error standard of the mean	0.6	0.7	0.5	0.5	0.5	0.5	0.6	0.2	0.6	0.6	0.4	0.4	0.4	0.4	0.5	0.2	0.1
Percentile 25 th	60.0	60.0	60.0	60.0	50.0	50.0	50.0	60.0	50.0	60.0	60.0	60.0	50.0	50.0	50.0	60.0	60.0
Median	70.0	70.0	70.0	70.0	70.0	70.0	60.0	70.0	70.0	70.0	70.0	70.0	70.0	70.0	70.0	70.0	70.0
Percentile 75th	80.0	80.0	80.0	80.0	80.0	80.0	80.0	80.0	80.0	80.0	80.0	80.0	80.0	80.0	80.0	80.0	80.0
Minimum	20.0	10.0	0.0	0.0	0.0	0.0	0.0	0.0	0.0	10.0	0.0	10.0	0.0	0.0	10.0	0.0	0.0
Maximum	100.0	100.0	100.0	100.0	100.0	100.0	100.0	100.0	100.0	100.0	100.0	100.0	100.0	100.0	100.0	100.0	100.0
Floor effect (%)	0.0	0.0	0.2	0.0	0.2	0.1	0.1	0.1	0.2	0.0	0.1	0.0	0.1	0.2	0.0	0.1	0.1
Ceiling effect (%)	6.0	5.1	4.7	3.5	3.8	3.5	4.0	4.3	2.8	4.7	4.7	5.4	4.6	6.1	5.2	4.9	4.6
																	
**General health**																	
n=	667	633	1384	1383	1285	1113	824	7289	725	805	1921	1883	1742	1682	1336	10094	17383
Mean	75.1	74.2	75.9	74.5	72.7	74.0	72.1	74.1	68.2	68.6	70.2	69.1	67.3	67.9	67.1	68.5	71.3
Standard deviation	14.4	16.4	13.3	13.7	14.7	12.5	14.8	14.1	15.2	15.3	13.3	14.7	14.4	12.4	13.6	14.0	14.3
Error standard of the mean	0.5	0.5	0.3	0.4	0.4	0.4	0.5	0.2	0.5	0.5	0.3	0.4	0.4	0.4	0.4	0.1	0.1
Percentile 25 th	70.0	66.7	70.0	66.7	63.3	66.7	63.3	66.7	60.0	60.0	60.0	60.0	56.7	56.7	56.7	60.0	63.3
Median	76.7	76.7	76.7	76.7	73.3	76.7	73.3	76.7	70.0	70.0	73.3	70.0	66.7	70.0	70.0	70.0	73.3
Percentile 75th	83.3	83.3	83.3	83.3	83.3	83.3	83.3	83.3	80.0	80.0	80.0	80.0	76.7	80.0	76.7	80.0	80.0
Minimum	26.7	23.3	20.0	16.7	13.3	16.7	26.7	13.3	20.0	20.0	16.7	10.0	6.7	0.0	10.0	0.0	0.0
Maximum	100.0	100.0	100.0	100.0	100.0	100.0	100.0	100.0	96.7	100.0	100.0	100.0	100.0	100.0	100.0	100.0	100.0
Floor effect (%)	0.0	0.0	-0.0	0.0	-0.0	0.0	0.0	0.0	0.0	0.0	0.0	0.0	0.0	0.1	0.0	0.0	0.0
Ceiling effect (%)	0.7	0.5	0.7	0.6	0.4	0.8	0.5	0.6	0.0	0.4	0.5	0.2	0.4	0.5	0.3	0.4	0.5
																	
**Perceived health**																	
n=	669	638	1399	1403	1317	1121	864	7411	735	810	1925	1919	1770	1728	1403	10290	17701
Mean	71.4	75.4	78.6	76.0	69.9	67.1	64.5	72.4	69.4	72.1	74.8	73.2	69.6	67.0	61.5	70.2	71.3
Standard deviation	43.5	44.5	33.5	34.0	37.0	31.5	37.5	36.8	39.3	38.9	32.4	32.4	31.3	27.1	29.5	32.3	34.3
Error standard of the mean	1.4	1.4	0.9	0.9	0.9	1.0	1.2	0.4	1.3	1.3	0.8	0.8	0.8	0.8	0.9	0.3	0.3
Percentile 25 th	50.0	50.0	50.0	50.0	50.0	50.0	50.0	50.0	50.0	50.0	50.0	50.0	50.0	50.0	50.0	50.0	50.0
Median	100.0	100.0	100.0	100.0	100.0	50.0	50.0	100.0	100.0	100.0	100.0	100.0	50.0	50.0	50.0	100.0	100.0
Percentile 75th	100.0	100.0	100.0	100.0	100.0	100.0	100.0	100.0	100.0	100.0	100.0	100.0	100.0	100.0	100.0	100.0	100.0
Minimum	0.0	0.0	0.0	0.0	0.0	0.0	0.0	0.0	0.0	0.0	0.0	0.0	0.0	0.0	0.0	0.0	0.0
Maximum	100.0	100.0	100.0	100.0	100.0	100.0	100.0	100.0	100.0	100.0	100.0	100.0	100.0	100.0	100.0	100.0	100.0
Floor effect (%)	13.1	11.9	8.0	8.7	11.1	11.7	13.2	10.8	14.5	13.8	11.3	10.4	10.2	12.3	13.5	11.9	11.3
Ceiling effect (%)	55.9	62.8	65.3	60.7	50.9	45.8	42.3	55.6	53.2	58.1	60.9	56.7	49.4	46.3	36.5	52.3	53.9
																	
**Self-esteem**																	
n=	669	635	1389	1387	1295	1120	838	7333	728	807	1924	1897	1754	1697	1372	10179	17512
Mean	77.1	76.2	80.2	79.4	79.3	78.7	79.3	78.8	68.2	70.6	75.7	75.2	74.8	75.6	77.2	74.4	76.5
Standard deviation	20.6	23.7	17.7	17.8	18.0	15.8	17.7	18.4	20.0	19.7	16.4	17.4	17.6	14.6	16.2	17.2	17.9
Error standard of the mean	0.7	0.7	0.5	0.5	0.5	0.5	0.6	0.2	0.7	0.6	0.4	0.4	0.4	0.5	0.5	0.2	0.1
Percentile 25 th	70.0	70.0	70.0	70.0	70.0	70.0	70.0	70.0	60.0	60.0	60.0	60.0	60.0	60.0	70.0	60.0	70.0
Median	80.0	80.0	80.0	80.0	80.0	80.0	80.0	80.0	70.0	70.0	80.0	80.0	80.0	80.0	80.0	80.0	80.0
Percentile 75th	90.0	90.0	90.0	90.0	90.0	90.0	90.0	90.0	80.0	80.0	90.0	90.0	90.0	90.0	90.0	90.0	90.0
Minimum	20.0	10.0	0.0	0.0	10.0	0.0	10.0	0.0	0.0	0.0	10.0	0.0	0.0	0.0	10.0	0.0	0.0
Maximum	100.0	100.0	100.0	100.0	100.0	100.0	100.0	100.0	100.0	100.0	100.0	100.0	100.0	100.0	100.0	100.0	100.0
Floor effect (%)	0.0	0.0	0.1	0.1	0.0	0.0	0.0	0.0	0.2	0.1	0.0	0.1	0.2	0.1	0.0	0.1	0.1
Ceiling effect (%)	15.2	16.0	20.6	20.0	19.4	19.2	20.5	18.9	5.6	7.0	13.6	14.3	14.2	15.7	18.2	13.2	16.0
																	
**Anxiety**																	
n=	669	636	1394	1395	1300	1116	845	7355	731	808	1924	1898	1756	1703	1381	10201	17556
Mean	29.2	32.1	28.3	28.3	28.5	24.4	24.5	28.0	35.9	37.4	34.1	33.5	34.1	31.6	30.0	33.7	30.9
Standard deviation	22.5	23.1	18.9	18.8	19.6	16.2	17.5	19.4	21.0	20.0	16.9	18.2	17.7	15.3	16.5	17.7	18.6
Error standard of the mean	0.7	0.7	0.5	0.5	0.5	0.5	0.6	0.2	0.7	0.7	0.4	0.4	0.4	0.5	0.5	0.2	0.1
Percentile 25 th	16.7	16.7	16.7	16.7	16.7	8.3	16.7	16.7	25.0	25.0	25.0	16.7	16.7	16.7	16.7	16.7	16.7
Median	25.0	33.3	25.0	25.0	25.0	25.0	25.0	25.0	33.3	33.3	33.3	33.3	33.3	33.3	25.0	33.3	33.3
Percentile 75th	41.7	41.7	41.7	41.7	41.7	33.3	33.3	41.7	50.0	50.0	50.0	41.7	50.0	41.7	41.7	50.0	41.7
Minimum	0.0	0.0	0.0	0.0	0.0	0.0	0.0	0.0	0.0	0.0	0.0	0.0	0.0	0.0	0.0	0.0	0.0
Maximum	83.3	91.7	100.0	91.7	91.7	91.7	83.3	100.0	91.7	91.7	91.7	100.0	100.0	100.0	100.0	100.0	100.0
Floor effect (%)	5.6	4.3	8.0	8.0	7.5	12.7	10.5	8.0	4.4	1.5	4.0	4.1	5.2	7.8	7.4	4.9	6.4
Ceiling effect (%)	0.0	0.0	0.1	0.0	-0.0	0.0	0.0	0.0	0.0	0.0	0.0	0.1	0.3	0.1	0.1	0.1	0.1
																	
**Depression**																	
n=	670	637	1398	1403	1313	1121	856	7398	736	810	1926	1915	1767	1721	1398	10273	17671
Mean	28.6	29.1	22.2	22.0	23.1	19.5	21.4	23.5	36.3	34.3	30.6	30.6	31.7	30.2	29.5	31.5	27.6
Standard deviation	24.2	24.6	19.1	18.3	20.3	16.8	19.5	20.2	23.6	22.3	19.4	20.2	20.0	17.1	18.9	19.9	20.4
Error standard of the mean	0.8	0.8	0.5	0.5	0.5	0.5	0.6	0.2	0.8	0.7	0.5	0.5	0.5	0.5	0.6	0.2	0.2
Percentile 25 th	10.0	10.0	10.0	10.0	10.0	10.0	10.0	10.0	20.0	20.0	20.0	20.0	20.0	10.0	10.0	20.0	10.0
Median	30.0	30.0	20.0	20.0	20.0	20.0	20.0	20.0	30.0	30.0	30.0	30.0	30.0	30.0	30.0	30.0	20.0
Percentile 75th	40.0	40.0	30.0	30.0	30.0	30.0	30.0	30.0	50.0	50.0	40.0	40.0	40.0	40.0	40.0	40.0	40.0
Minimum	0.0	0.0	0.0	0.0	0.0	0.0	0.0	0.0	0.0	0.0	0.0	0.0	0.0	0.0	0.0	0.0	0.0
Maximum	100.0	90.0	100.0	100.0	100.0	100.0	80.0	100.0	100.0	100.0	100.0	100.0	100.0	100.0	100.0	100.0	100.0
Floor effect (%)	9.3	8.7	15.9	15.8	17.4	23.8	18.6	15.7	7.3	5.9	8.5	9.2	9.1	11.9	11.2	9.1	12.4
Ceiling effect (%)	0.3	0.0	0.1	0.1	0.0	0.1	0.0	0.1	0.6	0.3	0.5	0.1	0.7	0.3	0.7	0.4	0.3
																	
**Pain**																	
n=	670	638	1398	1404	1320	1124	867	7421	736	810	1927	1919	1775	1729	1406	10302	17723
Mean	22.6	25.7	26.3	29.3	35.3	33.2	39.0	30.2	29.4	32.4	33.8	36.2	41.8	45.3	47.9	38.2	34.3
Standard deviation	37.4	41.5	32.8	33.3	37.3	30.5	36.9	35.6	34.0	37.7	32.8	33.4	33.8	28.9	32.3	33.4	34.5
Error standard of the mean	1.2	1.3	0.8	0.9	0.9	1.0	1.1	0.4	1.2	1.2	0.8	0.8	0.9	0.9	1.0	0.4	0.3
Percentile 25 th	0.0	0.0	0.0	0.0	0.0	0.0	0.0	0.0	0.0	0.0	0.0	0.0	0.0	0.0	0.0	0.0	0.0
Median	0.0	0.0	0.0	50.0	50.0	50.0	50.0	50.0	50.0	50.0	50.0	50.0	50.0	50.0	50.0	50.0	50.0
Percentile 75th	50.0	50.0	50.0	50.0	50.0	50.0	50.0	50.0	50.0	50.0	50.0	50.0	50.0	50.0	50.0	50.0	50.0
Minimum	0.0	0.0	0.0	0.0	0.0	0.0	0.0	0.0	0.0	0.0	0.0	0.0	0.0	0.0	0.0	0.0	0.0
Maximum	100.0	100.0	100.0	100.0	100.0	100.0	100.0	100.0	100.0	100.0	100.0	100.0	100.0	100.0	100.0	100.0	100.0
Floor effect (%)	61.1	57.4	54.5	49.7	42.5	43.9	36.3	49.2	48.9	48.4	45.9	42.0	35.4	31.9	28.8	40.1	44.6
Ceiling effect (%)	6.4	8.7	7.0	8.2	13.0	10.3	14.3	9.7	7.6	13.2	13.5	14.3	18.9	22.5	24.6	16.4	13.1
																	
**Disability**																	
n=	670	638	1399	1403	1319	1125	867	7421	737	810	1926	1919	1775	1730	1407	10304	17725
Mean	2.7	2.3	2.4	3.3	2.7	2.2	3.9	2.8	2.7	3.2	4.6	3.6	3.5	3.1	2.7	3.5	3.1
Standard deviation	15.6	15.9	13.8	16.4	15.8	12.2	19.5	15.6	14.2	14.4	17.0	16.0	15.8	12.3	12.5	14.9	15.2
Error standard of the mean	0.5	0.5	0.4	0.4	0.4	0.4	0.6	0.2	0.5	0.5	0.4	0.4	0.4	0.4	0.4	0.2	0.1
Percentile 25 th	0.0	0.0	0.0	0.0	0.0	0.0	0.0	0.0	0.0	0.0	0.0	0.0	0.0	0.0	0.0	0.0	0.0
Median	0.0	0.0	0.0	0.0	0.0	0.0	0.0	0.0	0.0	0.0	0.0	0.0	0.0	0.0	0.0	0.0	0.0
Percentile 75th	0.0	0.0	0.0	0.0	0.0	0.0	0.0	0.0	0.0	0.0	0.0	0.0	0.0	0.0	0.0	0.0	0.0
Minimum	0.0	0.0	0.0	0.0	0.0	0.0	0.0	0.0	0.0	0.0	0.0	0.0	0.0	0.0	0.0	0.0	0.0
Maximum	100.0	100.0	100.0	100.0	100.0	100.0	100.0	100.0	100.0	100.0	100.0	100.0	100.0	100.0	100.0	100.0	100.0
Floor effect (%)	95.4	96.3	96.5	95.1	96.3	96.9	95.1	96.0	95.5	94.2	93.3	94.9	95.3	95.7	96.2	94.9	95.4
Ceiling effect (%)	0.7	0.9	1.2	1.8	1.7	1.3	2.8	1.5	0.9	0.6	2.4	2.2	2.4	1.9	1.5	1.9	1.7

*Weighted by number of eligible persons in the household and imputed from 1999 INSEE National Census data.

Mean disability, depression, self-esteem and mental health scores were low for men (2.8 ± 15.6, 21.4 ± 19.5, 78.8 ± 18.4, 78.6 ± 19.6, respectively), and disability, mental health, self-esteem and physical health scores were low for women (3.5 ± 14.9, 74.6 ± 20.2, 74.4 ± 17.2, 72.3 ± 20.3, respectively). The most affected dimension was social health for men (64.4 ± 19.2) and pain for women (38.31 ± 33.4).

Tables 4 and 5 provide the HRQoL norms by gender, age and self-reported chronic disease. Self-reported chronic disease was associated with a mean decrease of 12.5 points in the score for physical health, 4.6 for mental health, 2.3 for social health, 6.5 for general health, 19.3 for perceived health, and 3.2 for self-esteem and a mean increase of 4.8 points in the score for anxiety, 5.1 for depression, 18.4 for pain and 2.5 for disability (for the last 4 dimensions, the interpretation of the score is inversed). All differences were statistically significant (p < 0.001), whatever the gender and age. After adjustment for gender and age, significant interactions were found between self-reported chronic disease and age for perceived health (p < 0.0001) and depression (p < 0.0001): increase in age had a lower effect on HRQoL score in the group with a self-reported chronic disease. We also observed a significant interaction between gender and self-reported chronic disease, with greater effects for women than men in score for physical health (-14 points and -11 points, respectively, p = 0.003), general health (-7 points and -5.7 points, respectively, p = 0.002), and pain (+23.6 points and 15.9 points, respectively, p = 0.001).

**Table 4:** HRQoL norms* in French general population from 12 to 75 years old with self-reported chronic disease (n = 4,153)

	**Men**								**Women**								**Total**
			
	**12-17**	**18-24**	**25-34**	**35-44**	**45-54**	**55-64**	**65-75**	**Total**	**12-17**	**18-24**	**25-34**	**35-44**	**45-54**	**55-64**	**65-75**	**Total**	
																
																	
																	
**Physical health**																	
n=	62	44	177	222	329	375	402	1611	55	105	276	338	489	631	625	2519	4130
Mean	74.2	72.1	69.2	70.1	66.5	68.3	65.7	68.0	60.0	61.9	63.4	57.2	55.9	57.3	55.4	57.6	62.5
Standard deviation	23.7	20.8	21.4	20.2	25.2	19.6	23.7	22.4	21.8	21.7	20.1	23.5	20.4	18.2	19.6	20.3	21.7
Error standard of the mean	2.5	2.4	1.6	1.3	1.3	1.1	1.1	0.5	2.7	2.0	1.3	1.3	1.0	0.9	0.9	0.4	0.4
Percentile 25 th	60.0	60.0	60.0	60.0	50.0	60.0	50.0	60.0	50.0	50.0	50.0	40.0	40.0	40.0	40.0	40.0	50.0
Median	80.0	70.0	70.0	70.0	70.0	70.0	70.0	70.0	60.0	60.0	70.0	60.0	60.0	60.0	60.0	60.0	70.0
Percentile 75th	90.0	80.0	80.0	80.0	80.0	80.0	80.0	80.0	70.0	80.0	80.0	80.0	70.0	70.0	70.0	70.0	80.0
Minimum	20.0	30.0	0.0	0.0	0.0	0.0	0.0	0.0	20.0	10.0	10.0	0.0	0.0	0.0	0.0	0.0	0.0
Maximum	100.0	100.0	100.0	100.0	100.0	100.0	100.0	100.0	100.0	100.0	100.0	100.0	100.0	100.0	100.0	100.0	100.0
Floor effect (%)	0.0	0.0	0.5	0.8	1.4	0.6	1.6	1.0	0.0	0.0	0.0	2.5	1.8	1.1	1.8	1.4	1.2
Ceiling effect (%)	16.2	7.7	9.1	3.3	6.7	9.2	4.5	6.9	4.2	3.7	4.4	2.4	2.5	3.4	1.2	2.7	4.6
																	
**Mental health**																	
n=	62	44	178	221	329	376	401	1611	55	105	276	338	486	628	620	2508	4119
Mean	67.9	73.8	74.4	73.4	74.6	78.2	79.1	75.9	61.8	65.7	67.4	65.0	63.2	68.3	70.4	66.8	71.0
Standard deviation	26.5	23.3	21.4	19.6	20.6	17.2	18.2	19.8	24.0	24.6	21.0	23.1	21.3	17.4	19.3	20.5	20.7
Error standard of the mean	2.8	2.7	1.6	1.3	1.1	0.9	0.8	0.5	3.0	2.2	1.4	1.3	1.0	0.9	0.9	0.5	0.3
Percentile 25 th	50.0	60.0	60.0	60.0	60.0	70.0	70.0	60.0	50.0	50.0	50.0	50.0	50.0	60.0	60.0	50.0	60.0
Median	70.0	80.0	80.0	80.0	80.0	80.0	80.0	80.0	60.0	70.0	70.0	70.0	70.0	70.0	70.0	70.0	70.0
Percentile 75th	90.0	90.0	90.0	90.0	90.0	90.0	90.0	90.0	80.0	80.0	80.0	80.0	80.0	90.0	90.0	80.0	90.0
Minimum	20.0	20.0	0.0	0.0	10.0	0.0	10.0	0.0	10.0	10.0	0.0	0.0	0.0	0.0	0.0	0.0	0.0
Maximum	100.0	100.0	100.0	100.0	100.0	100.0	100.0	100.0	100.0	100.0	100.0	100.0	100.0	100.0	100.0	100.0	100.0
Floor effect (%)	0.0	0.0	1.3	0.3	0.0	0.2	0.0	0.2	0.0	0.0	1.5	0.1	1.6	0.7	1.0	0.9	0.6
Ceiling effect (%)	9.1	8.7	13.1	12.3	14.9	21.2	17.7	15.7	6.0	7.4	7.0	8.2	7.8	8.4	10.9	8.5	11.9
																	
**Social health**																	
n=	62	45	176	217	327	374	392	1593	55	105	276	336	486	623	617	2498	4091
Mean	67.9	69.6	69.0	65.4	64.3	64.5	63.1	65.1	66.2	64.8	68.9	63.7	63.9	65.8	64.0	65.0	65.0
Standard deviation	22.0	23.8	19.4	19.7	19.4	17.1	19.9	19.4	19.6	22.6	15.9	18.8	17.4	15.2	16.6	17.1	18.0
Error standard of the mean	2.3	2.8	1.4	1.3	1.0	0.9	0.9	0.5	2.4	2.0	1.0	1.0	0.9	0.8	0.8	0.4	0.3
Percentile 25 th	60.0	60.0	60.0	50.0	50.0	50.0	50.0	50.0	60.0	50.0	60.0	50.0	50.0	50.0	50.0	50.0	50.0
Median	70.0	70.0	70.0	60.0	60.0	70.0	60.0	70.0	70.0	70.0	70.0	60.0	70.0	70.0	70.0	70.0	70.0
Percentile 75th	80.0	80.0	80.0	80.0	80.0	80.0	80.0	80.0	80.0	80.0	80.0	80.0	80.0	80.0	80.0	80.0	80.0
Minimum	20.0	10.0	0.0	10.0	0.0	0.0	10.0	0.0	0.0	10.0	20.0	10.0	0.0	0.0	10.0	0.0	0.0
Maximum	100.0	100.0	100.0	100.0	100.0	100.0	100.0	100.0	90.0	100.0	100.0	100.0	100.0	100.0	100.0	100.0	100.0
Floor effect (%)	0.0	0.0	0.4	0.0	0.5	0.4	0.0	0.2	2.5	0.0	0.0	0.0	0.5	0.1	0.0	0.2	0.2
Ceiling effect (%)	8.9	11.7	6.9	5.4	4.8	2.1	4.3	5.0	0.0	8.4	4.9	4.2	4.9	5.9	5.2	5.1	5.0
																	
**General health**																	
n=	62	44	175	216	324	374	385	1580	55	105	276	333	481	614	599	2463	4043
Mean	70.0	71.9	71.0	69.7	68.4	70.3	69.2	69.6	62.7	64.2	66.6	62.0	61.0	63.8	63.3	63.1	66.2
Standard deviation	17.7	14.5	15.3	15.0	16.0	13.0	14.5	14.8	15.7	16.5	14.0	17.3	15.1	12.6	13.6	14.5	14.9
Error standard of the mean	1.9	1.7	1.1	1.0	0.8	0.7	0.7	0.4	1.9	1.5	0.9	1.0	0.7	0.6	0.6	0.3	0.2
Percentile 25 th	60.0	66.7	63.3	60.0	56.7	63.3	60.0	60.0	53.3	53.3	56.7	50.0	50.0	53.3	53.3	53.3	56.7
Median	70.0	73.3	73.3	73.3	70.0	73.3	70.0	73.3	63.3	66.7	70.0	63.3	63.3	66.7	63.3	63.3	66.7
Percentile 75th	80.0	80.0	83.3	80.0	80.0	80.0	80.0	80.0	73.3	76.7	76.7	73.3	73.3	76.7	73.3	73.3	76.7
Minimum	36.7	26.7	20.0	30.0	13.3	16.7	26.7	13.3	26.7	23.3	16.7	10.0	6.7	16.7	10.0	6.7	6.7
Maximum	93.3	86.7	96.7	100.0	96.7	100.0	96.7	100.0	86.7	93.3	96.7	96.7	100.0	96.7	96.7	100.0	100.0
Floor effect (%)	0.0	0.0	0.0	0.0	0.0	0.0	0.0	0.0	0.0	0.0	0.0	0.0	0.0	0.0	0.0	0.0	0.0
Ceiling effect (%)	0.0	0.0	0.0	0.8	0.0	0.3	0.0	0.2	0.0	0.0	0.0	0.0	0.2	0.0	0.0	0.0	0.1
																	
**Perceived health**																	
n=	62	45	178	222	330	374	403	1614	55	105	275	340	489	633	631	2528	4142
Mean	70.8	80.5	66.6	59.9	53.9	51.5	52.0	57.0	66.9	60.6	62.7	57.6	54.0	53.3	50.3	55.4	56.2
Standard deviation	40.1	34.4	34.0	39.9	38.7	32.7	36.5	37.2	39.2	43.2	34.3	35.8	33.0	27.4	28.3	32.0	34.1
Error standard of the mean	4.3	4.0	2.5	2.5	2.0	1.8	1.7	0.9	4.8	3.9	2.2	2.0	1.6	1.4	1.3	0.7	0.6
Percentile 25 th	50.0	50.0	50.0	50.0	50.0	50.0	50.0	50.0	50.0	50.0	50.0	50.0	50.0	50.0	50.0	50.0	50.0
Median	100.0	100.0	50.0	50.0	50.0	50.0	50.0	50.0	50.0	50.0	50.0	50.0	50.0	50.0	50.0	50.0	50.0
Percentile 75th	100.0	100.0	100.0	100.0	100.0	100.0	50.0	100.0	100.0	100.0	100.0	100.0	100.0	100.0	50.0	100.0	100.0
Minimum	0.0	0.0	0.0	0.0	0.0	0.0	0.0	0.0	0.0	0.0	0.0	0.0	0.0	0.0	0.0	0.0	0.0
Maximum	100.0	100.0	100.0	100.0	100.0	100.0	100.0	100.0	100.0	100.0	100.0	100.0	100.0	100.0	100.0	100.0	100.0
Floor effect (%)	10.1	2.0	11.0	20.5	22.7	22.8	20.2	19.0	13.9	23.4	17.5	20.1	21.9	20.9	20.8	20.5	19.8
Ceiling effect (%)	51.6	63.0	44.2	40.4	30.4	25.8	24.3	33.1	47.6	44.5	42.8	35.4	30.0	27.6	21.4	31.3	32.2
																	
**Self-esteem**																	
n=	62	45	176	217	327	375	391	1593	55	105	276	335	483	619	612	2485	4078
Mean	71.7	77.5	77.8	74.5	77.4	76.2	77.5	76.6	66.8	68.6	73.1	70.5	70.3	72.7	73.8	71.7	74.0
Standard deviation	20.7	21.4	18.5	19.1	19.5	16.8	17.9	18.5	20.4	22.3	16.4	19.4	19.0	14.9	16.4	17.4	18.0
Error standard of the mean	2.2	2.5	1.4	1.2	1.0	0.9	0.8	0.4	2.5	2.0	1.1	1.1	0.9	0.8	0.8	0.4	0.3
Percentile 25 th	60.0	70.0	70.0	60.0	70.0	70.0	70.0	70.0	60.0	60.0	60.0	60.0	60.0	60.0	60.0	60.0	60.0
Median	80.0	80.0	80.0	80.0	80.0	80.0	80.0	80.0	70.0	70.0	80.0	70.0	70.0	80.0	80.0	70.0	80.0
Percentile 75th	90.0	90.0	90.0	90.0	90.0	90.0	90.0	90.0	80.0	90.0	90.0	80.0	90.0	90.0	90.0	90.0	90.0
Minimum	40.0	10.0	20.0	0.0	10.0	0.0	10.0	0.0	10.0	10.0	20.0	0.0	0.0	0.0	20.0	0.0	0.0
Maximum	100.0	100.0	100.0	100.0	100.0	100.0	100.0	100.0	100.0	100.0	100.0	100.0	100.0	100.0	100.0	100.0	100.0
Floor effect (%)	0.0	0.0	0.0	0.2	0.0	0.1	0.0	0.0	0.0	0.0	0.0	0.1	0.8	0.1	0.0	0.2	0.1
Ceiling effect (%)	6.4	12.4	17.2	14.2	17.8	14.7	15.0	15.1	2.9	10.5	10.6	7.1	10.4	11.2	13.0	10.4	12.6
																	
**Anxiety**																	
n=	62	44	177	220	328	375	395	1601	55	105	276	336	486	626	620	2504	4105
Mean	36.2	35.7	35.0	34.5	32.1	28.2	26.5	30.9	43.5	41.8	38.8	41.8	40.5	35.8	33.2	38.1	34.7
Standard deviation	25.3	22.7	19.9	20.5	20.2	17.3	17.1	19.4	21.5	20.7	17.4	19.8	17.4	15.5	16.9	17.7	18.7
Error standard of the mean	2.7	2.6	1.5	1.3	1.0	1.0	0.8	0.5	2.6	1.9	1.1	1.1	0.9	0.8	0.8	0.4	0.3
Percentile 25 th	25.0	25.0	25.0	16.7	16.7	16.7	16.7	16.7	33.3	25.0	25.0	25.0	25.0	25.0	16.7	25.0	16.7
Median	33.3	41.7	33.3	33.3	33.3	25.0	25.0	25.0	41.7	41.7	41.7	41.7	41.7	33.3	33.3	33.3	33.3
Percentile 75th	50.0	41.7	50.0	50.0	41.7	41.7	33.3	41.7	50.0	58.3	50.0	50.0	50.0	50.0	41.7	50.0	50.0
Minimum	0.0	8.3	0.0	0.0	0.0	0.0	0.0	0.0	0.0	8.3	0.0	0.0	0.0	0.0	0.0	0.0	0.0
Maximum	83.3	75.0	100.0	91.7	91.7	91.7	83.3	100.0	91.7	91.7	91.7	100.0	100.0	91.7	91.7	100.0	100.0
Floor effect (%)	3.3	0.0	3.1	3.9	5.6	7.1	6.7	5.3	4.4	0.0	1.2	0.6	2.0	4.9	6.2	3.2	4.2
Ceiling effect (%)	0.0	0.0	0.8	0.0	0.0	0.0	0.0	0.1	0.0	0.0	0.0	0.6	0.7	0.0	0.0	0.2	0.2
																	
**Depression**																	
n=	62	44	178	222	330	376	400	1612	55	105	276	339	487	631	627	2520	4132
Mean	35.7	33.3	28.7	27.1	27.7	23.6	23.1	26.3	44.9	37.1	35.5	38.7	39.1	34.0	32.9	36.2	31.6
Standard deviation	30.5	26.5	21.3	19.4	22.0	17.9	19.4	20.9	26.2	24.2	21.0	22.2	20.2	17.2	19.4	20.2	21.0
Error standard of the mean	3.2	3.1	1.6	1.2	1.1	1.0	0.9	0.5	3.2	2.2	1.4	1.2	1.0	0.9	0.9	0.4	0.3
Percentile 25 th	20.0	20.0	10.0	10.0	10.0	10.0	10.0	10.0	30.0	20.0	20.0	20.0	20.0	20.0	20.0	20.0	20.0
Median	30.0	30.0	30.0	30.0	20.0	20.0	20.0	20.0	50.0	30.0	30.0	40.0	40.0	30.0	30.0	30.0	30.0
Percentile 75th	50.0	50.0	40.0	40.0	40.0	40.0	30.0	40.0	60.0	50.0	50.0	60.0	50.0	50.0	50.0	50.0	50.0
Minimum	0.0	0.0	0.0	0.0	0.0	0.0	0.0	0.0	0.0	0.0	0.0	0.0	0.0	0.0	0.0	0.0	0.0
Maximum	100.0	80.0	100.0	100.0	100.0	90.0	80.0	100.0	100.0	100.0	100.0	100.0	100.0	100.0	100.0	100.0	100.0
Floor effect (%)	7.7	6.4	9.6	8.4	13.3	16.4	13.9	12.4	4.4	5.6	5.0	3.7	2.8	7.1	8.9	5.7	8.8
Ceiling effect (%)	3.4	0.0	0.4	0.5	0.2	0.0	0.0	0.3	1.5	1.7	2.1	0.3	1.6	0.3	1.4	1.2	0.8
																	
**Pain**																	
n=	62	45	177	222	331	375	404	1616	55	105	276	340	490	635	632	2533	4149
Mean	34.5	39.9	41.0	41.2	44.9	43.0	44.6	42.8	41.2	53.7	46.5	52.3	56.1	55.9	56.8	53.9	48.7
Standard deviation	39.2	46.8	37.7	34.2	39.5	32.4	38.1	36.9	36.5	39.6	34.8	36.9	34.8	29.2	32.3	33.6	35.3
Error standard of the mean	4.2	5.4	2.8	2.2	2.0	1.8	1.7	0.9	4.5	3.6	2.2	2.0	1.7	1.5	1.5	0.7	0.6
Percentile 25 th	0.0	0.0	0.0	0.0	0.0	0.0	0.0	0.0	0.0	50.0	0.0	0.0	50.0	50.0	50.0	50.0	0.0
Median	50.0	50.0	50.0	50.0	50.0	50.0	50.0	50.0	50.0	50.0	50.0	50.0	50.0	50.0	50.0	50.0	50.0
Percentile 75th	50.0	50.0	50.0	50.0	50.0	50.0	50.0	50.0	50.0	100.0	50.0	100.0	100.0	100.0	100.0	100.0	100.0
Minimum	0.0	0.0	0.0	0.0	0.0	0.0	0.0	0.0	0.0	0.0	0.0	0.0	0.0	0.0	0.0	0.0	0.0
Maximum	100.0	100.0	100.0	100.0	100.0	100.0	100.0	100.0	100.0	100.0	100.0	100.0	100.0	100.0	100.0	100.0	100.0
Floor effect (%)	41.6	38.1	37.8	31.3	32.9	31.8	30.2	32.9	32.1	23.2	31.5	26.0	23.1	22.1	21.5	24.3	28.3
Ceiling effect (%)	10.6	17.8	19.8	13.7	22.7	17.8	19.5	18.6	14.5	30.6	24.4	30.6	35.4	34.0	35.1	32.0	25.8
																	
**Disability**																	
n=	62	45	178	222	331	376	404	1618	55	105	276	340	490	635	633	2534	4152
Mean	5.5	2.0	6.9	6.7	4.1	3.5	5.8	5.1	3.7	5.5	6.6	6.1	5.6	4.5	3.7	5.1	5.1
Standard deviation	20.5	12.8	22.6	23.4	19.2	15.1	24.2	20.7	14.5	19.1	21.0	20.2	19.1	14.8	14.7	17.4	18.7
Error standard of the mean	2.2	1.5	1.7	1.5	1.0	0.8	1.1	0.5	1.8	1.7	1.4	1.1	0.9	0.7	0.7	0.4	0.3
Percentile 25 th	0.0	0.0	0.0	0.0	0.0	0.0	0.0	0.0	0.0	0.0	0.0	0.0	0.0	0.0	0.0	0.0	0.0
Median	0.0	0.0	0.0	0.0	0.0	0.0	0.0	0.0	0.0	0.0	0.0	0.0	0.0	0.0	0.0	0.0	0.0
Percentile 75th	0.0	0.0	0.0	0.0	0.0	0.0	0.0	0.0	0.0	0.0	0.0	0.0	0.0	0.0	0.0	0.0	0.0
Minimum	0.0	0.0	0.0	0.0	0.0	0.0	0.0	0.0	0.0	0.0	0.0	0.0	0.0	0.0	0.0	0.0	0.0
Maximum	100.0	50.0	100.0	100.0	100.0	100.0	100.0	100.0	50.0	100.0	100.0	100.0	100.0	100.0	100.0	100.0	100.0
Floor effect (%)	90.0	95.9	90.1	90.8	94.6	95.0	92.9	93.1	92.6	90.3	91.1	90.8	92.5	93.8	94.8	92.7	92.9
Ceiling effect (%)	1.0	0.0	3.9	4.1	2.7	1.9	4.6	3.2	0.0	1.3	4.3	3.1	3.6	2.8	2.2	2.9	3.0

* Weighted by number of eligible persons in the household and imputed from 1999 INSEE National Census data.

**Table 5:** HRQoL norms* in French general population from 12 to 75 years old with no self-reported chronic disease (n = 13559)

	**Men**								**Women**								**Total**
			
	**12-17**	**18-24**	**25-34**	**35-44**	**45-54**	**55-64**	**65-75**	**Total**	**12-17**	**18-24**	**25-34**	**35-44**	**45-54**	**55-64**	**65-75**	**Total**	
																
																	
																	
**Physical health**																	
n=	608	593	1217	1180	985	745	460	5788	676	703	1648	1571	1279	1089	753	7719	13507
Mean	81.3	79.8	80.4	79.1	76.3	79.7	75.8	79.1	73.9	72.7	71.2	72.0	69.8	69.1	66.0	71.0	75.0
Standard deviation	18.4	19.8	16.5	17.2	20.5	16.3	21.3	18.4	19.6	20.0	18.4	18.2	18.8	16.9	18.4	18.6	19.0
Error standard of the mean	0.6	0.6	0.4	0.5	0.6	0.6	0.9	0.2	0.7	0.7	0.5	0.5	0.6	0.6	0.8	0.2	0.2
Percentile 25 th	70.0	70.0	70.0	70.0	70.0	70.0	70.0	70.0	60.0	60.0	60.0	60.0	60.0	60.0	50.0	60.0	60.0
Median	80.0	80.0	80.0	80.0	80.0	80.0	80.0	80.0	80.0	70.0	70.0	80.0	70.0	70.0	70.0	70.0	80.0
Percentile 75th	90.0	90.0	90.0	90.0	90.0	90.0	90.0	90.0	90.0	90.0	90.0	80.0	80.0	80.0	80.0	90.0	90.0
Minimum	20.0	0.0	10.0	0.0	0.0	0.0	0.0	0.0	0.0	0.0	0.0	0.0	0.0	0.0	0.0	0.0	0.0
Maximum	100.0	100.0	100.0	100.0	100.0	100.0	100.0	100.0	100.0	100.0	100.0	100.0	100.0	100.0	100.0	100.0	100.0
Floor effect (%)	0.0	0.3	0.0	0.1	0.6	0.2	0.4	0.2	0.4	0.2	0.5	0.3	0.4	0.7	0.6	0.4	0.3
Ceiling effect (%)	18.1	16.2	18.2	16.5	14.7	21.0	15.1	17.0	11.7	9.1	9.4	8.9	8.6	10.9	6.0	9.2	13.1
																	
**Mental health**																	
n=	606	593	1218	1179	981	745	454	5776	677	703	1646	1570	1278	1089	764	7727	13503
Mean	74.9	75.3	80.8	80.6	79.3	83.1	82.6	79.3	66.0	67.7	72.8	72.6	72.8	74.6	77.0	71.9	75.6
Standard deviation	22.1	24.7	17.8	17.9	19.2	16.1	18.9	19.4	23.4	21.7	18.9	19.5	19.1	16.6	17.8	19.6	19.9
Error standard of the mean	0.7	0.8	0.5	0.5	0.6	0.6	0.8	0.2	0.8	0.8	0.5	0.5	0.6	0.6	0.7	0.2	0.2
Percentile 25 th	60.0	60.0	70.0	70.0	70.0	70.0	70.0	70.0	50.0	50.0	60.0	60.0	60.0	60.0	70.0	60.0	60.0
Median	80.0	80.0	80.0	80.0	80.0	90.0	90.0	80.0	70.0	70.0	80.0	80.0	70.0	80.0	80.0	70.0	80.0
Percentile 75th	90.0	90.0	90.0	90.0	90.0	100.0	100.0	90.0	80.0	80.0	90.0	90.0	90.0	90.0	90.0	90.0	90.0
Minimum	0.0	0.0	20.0	10.0	20.0	10.0	20.0	0.0	0.0	0.0	0.0	0.0	0.0	0.0	0.0	0.0	0.0
Maximum	100.0	100.0	100.0	100.0	100.0	100.0	100.0	100.0	100.0	100.0	100.0	100.0	100.0	100.0	100.0	100.0	100.0
Floor effect (%)	0.1	0.1	0.0	0.0	0.0	0.0	0.0	0.0	0.5	0.2	0.3	0.1	0.5	0.4	0.2	0.3	0.2
Ceiling effect (%)	12.5	15.9	22.8	22.1	21.2	30.6	31.9	21.5	8.7	7.6	11.7	13.3	14.9	19.4	19.6	13.2	17.3
																	
**Social health**																	
n=	607	589	1210	1169	967	742	448	5732	674	701	1644	1564	1268	1075	758	7684	13416
Mean	70.5	68.0	68.7	66.6	66.1	65.2	65.5	67.4	65.6	67.0	68.6	67.8	66.1	67.2	67.1	67.2	67.3
Standard deviation	20.0	22.7	18.1	17.8	18.7	16.6	18.5	18.8	18.5	19.0	15.9	16.6	16.4	14.2	15.3	16.4	17.5
Error standard of the mean	0.7	0.7	0.5	0.5	0.5	0.7	0.8	0.2	0.7	0.7	0.4	0.4	0.5	0.6	0.6	0.2	0.1
Percentile 25 th	60.0	60.0	60.0	60.0	60.0	50.0	50.0	60.0	50.0	60.0	60.0	60.0	50.0	60.0	60.0	60.0	60.0
Median	70.0	70.0	70.0	70.0	70.0	70.0	70.0	70.0	70.0	70.0	70.0	70.0	70.0	70.0	70.0	70.0	70.0
Percentile 75th	80.0	80.0	80.0	80.0	80.0	80.0	80.0	80.0	80.0	80.0	80.0	80.0	80.0	80.0	80.0	80.0	80.0
Minimum	20.0	10.0	0.0	0.0	0.0	10.0	0.0	0.0	10.0	10.0	0.0	10.0	10.0	0.0	20.0	0.0	0.0
Maximum	100.0	100.0	100.0	100.0	100.0	100.0	100.0	100.0	100.0	100.0	100.0	100.0	100.0	100.0	100.0	100.0	100.0
Floor effect (%)	0.0	0.0	0.1	0.0	0.0	0.0	0.1	0.0	-0.0	0.0	0.1	0.0	0.0	0.2	-0.0	0.0	0.0
Ceiling effect (%)	5.7	4.5	4.5	3.1	3.5	4.2	3.7	4.1	3.0	4.1	4.6	5.7	4.5	6.2	5.1	4.8	4.5
																	
**General health**																	
n=	605	589	1207	1166	960	738	438	5703	669	699	1643	1549	1259	1067	734	7620	13323
Mean	75.6	74.4	76.6	75.4	74.0	76.0	74.6	75.3	68.6	69.2	70.8	70.8	69.6	70.3	70.2	70.1	72.7
Standard deviation	13.8	16.6	12.9	13.2	13.9	11.8	14.5	13.7	15.1	15.0	13.1	13.6	13.5	11.9	12.9	13.5	13.8
Error standard of the mean	0.5	0.5	0.3	0.4	0.4	0.5	0.6	0.2	0.5	0.5	0.3	0.4	0.4	0.5	0.5	0.2	0.1
Percentile 25 th	70.0	66.7	70.0	66.7	66.7	66.7	66.7	66.7	60.0	60.0	63.3	63.3	60.0	60.0	63.3	60.0	63.3
Median	76.7	76.7	76.7	76.7	73.3	76.7	76.7	76.7	70.0	70.0	73.3	73.3	70.0	73.3	73.3	70.0	73.3
Percentile 75th	83.3	83.3	86.7	86.7	83.3	86.7	83.3	83.3	80.0	80.0	80.0	80.0	80.0	80.0	80.0	80.0	83.3
Minimum	26.7	23.3	26.7	16.7	13.3	23.3	30.0	13.3	20.0	20.0	23.3	20.0	16.7	0.0	10.0	0.0	0.0
Maximum	100.0	100.0	100.0	100.0	100.0	100.0	100.0	100.0	96.7	100.0	100.0	100.0	100.0	100.0	100.0	100.0	100.0
Floor effect (%)	0.0	0.0	0.0	0.0	0.0	-0.0	0.0	0.0	0.0	0.0	0.0	0.0	0.0	0.1	0.0	0.0	0.0
Ceiling effect (%)	0.7	0.5	0.8	0.6	0.5	1.1	0.9	0.7	0.0	0.5	0.6	0.3	0.5	0.8	0.6	0.4	0.6
																	
**Perceived health**																	
n=	607	593	1219	1180	985	745	460	5789	678	704	1647	1578	1279	1094	769	7749	13538
Mean	71.4	75.0	80.3	79.1	75.1	75.2	75.6	76.4	69.5	73.9	76.8	76.7	75.3	75.0	70.4	74.6	75.5
Standard deviation	43.8	45.1	33.1	31.8	34.6	28.1	34.2	35.4	39.3	37.9	31.7	30.6	28.8	24.8	28.1	31.3	33.1
Error standard of the mean	1.5	1.5	0.9	0.9	1.0	1.1	1.5	0.4	1.4	1.3	0.8	0.8	0.8	0.9	1.1	0.4	0.3
Percentile 25 th	50.0	50.0	50.0	50.0	50.0	50.0	50.0	50.0	50.0	50.0	50.0	50.0	50.0	50.0	50.0	50.0	50.0
Median	100.0	100.0	100.0	100.0	100.0	100.0	100.0	100.0	100.0	100.0	100.0	100.0	100.0	100.0	50.0	100.0	100.0
Percentile 75th	100.0	100.0	100.0	100.0	100.0	100.0	100.0	100.0	100.0	100.0	100.0	100.0	100.0	100.0	100.0	100.0	100.0
Minimum	0.0	0.0	0.0	0.0	0.0	0.0	0.0	0.0	0.0	0.0	0.0	0.0	0.0	0.0	0.0	0.0	0.0
Maximum	100.0	100.0	100.0	100.0	100.0	100.0	100.0	100.0	100.0	100.0	100.0	100.0	100.0	100.0	100.0	100.0	100.0
Floor effect (%)	13.4	12.7	7.7	6.5	7.3	5.8	7.1	8.6	14.6	12.4	10.3	8.2	6.0	7.2	7.6	9.3	9.0
Ceiling effect (%)	56.3	62.8	68.2	64.6	57.5	56.2	58.2	61.4	53.6	60.1	63.9	61.6	56.5	57.1	48.5	58.5	60.0
																	
**Self-esteem**																	
n=	607	590	1211	1169	966	743	446	5732	672	701	1646	1561	1269	1077	757	7683	13415
Mean	77.6	76.2	80.5	80.4	79.8	79.9	80.9	79.4	68.2	70.9	76.1	76.3	76.4	77.2	80.0	75.1	77.2
Standard deviation	20.5	23.9	17.5	17.4	17.4	15.2	17.5	18.4	20.0	19.3	16.3	16.8	16.8	14.4	15.5	17.1	17.8
Error standard of the mean	0.7	0.8	0.5	0.5	0.5	0.6	0.8	0.2	0.7	0.7	0.4	0.4	0.5	0.6	0.6	0.2	0.2
Percentile 25 th	70.0	70.0	70.0	70.0	70.0	70.0	70.0	70.0	60.0	60.0	70.0	70.0	70.0	70.0	70.0	60.0	70.0
Median	80.0	80.0	80.0	80.0	80.0	80.0	80.0	80.0	70.0	70.0	80.0	80.0	80.0	80.0	80.0	80.0	80.0
Percentile 75th	90.0	90.0	90.0	90.0	90.0	90.0	100.0	90.0	80.0	80.0	90.0	90.0	90.0	90.0	90.0	90.0	90.0
Minimum	20.0	10.0	0.0	0.0	10.0	30.0	10.0	0.0	0.0	0.0	10.0	0.0	10.0	0.0	10.0	0.0	0.0
Maximum	100.0	100.0	100.0	100.0	100.0	100.0	100.0	100.0	100.0	100.0	100.0	100.0	100.0	100.0	100.0	100.0	100.0
Floor effect (%)	0.0	0.0	0.1	0.1	0.0	-0.0	0.0	0.0	0.2	0.1	0.0	0.1	0.0	0.1	0.0	0.1	0.0
Ceiling effect (%)	16.1	16.3	21.1	21.1	19.8	21.6	25.4	19.9	5.8	6.5	14.1	15.9	15.6	18.4	22.3	14.0	17.0
																	
**Anxiety**																	
n=	607	592	1215	1174	971	740	449	5748	674	702	1645	1561	1268	1076	758	7684	13432
Mean	28.6	31.8	27.4	27.1	27.3	22.4	22.6	27.3	35.3	36.8	33.3	31.7	31.8	29.1	27.5	32.4	29.9
Standard deviation	22.1	23.1	18.6	18.3	19.2	15.3	17.7	19.3	20.9	19.8	16.7	17.4	17.3	14.8	15.8	17.5	18.5
Error standard of the mean	0.7	0.7	0.5	0.5	0.6	0.6	0.8	0.2	0.7	0.7	0.4	0.5	0.5	0.6	0.6	0.2	0.2
Percentile 25 th	16.7	16.7	16.7	16.7	16.7	8.3	8.3	16.7	25.0	25.0	25.0	16.7	16.7	16.7	16.7	16.7	16.7
Median	25.0	33.3	25.0	25.0	25.0	16.7	16.7	25.0	33.3	33.3	33.3	33.3	33.3	25.0	25.0	33.3	25.0
Percentile 75th	41.7	41.7	41.7	41.7	41.7	33.3	33.3	41.7	50.0	50.0	41.7	41.7	41.7	41.7	41.7	41.7	41.7
Minimum	0.0	0.0	0.0	0.0	0.0	0.0	0.0	0.0	0.0	0.0	0.0	0.0	0.0	0.0	0.0	0.0	0.0
Maximum	83.3	91.7	91.7	91.7	91.7	83.3	75.0	91.7	91.7	83.3	91.7	91.7	100.0	100.0	100.0	100.0	100.0
Floor effect (%)	5.8	4.6	8.7	8.8	8.1	15.7	13.9	8.7	4.4	1.8	4.4	4.9	6.4	9.4	8.3	5.4	7.0
Ceiling effect (%)	0.0	0.0	0.0	0.0	0.0	0.0	0.0	0.0	0.0	0.0	0.0	0.0	0.1	0.1	0.1	0.0	0.0
																	
**Depression**																	
n=	608	593	1218	1180	982	744	455	5780	679	704	1647	1575	1278	1089	768	7740	13520
Mean	27.9	28.8	21.3	21.0	21.7	17.4	19.8	22.8	35.6	33.9	29.7	28.8	29.0	27.9	26.8	30.1	26.5
Standard deviation	23.3	24.5	18.6	18.0	19.4	15.9	19.6	20.0	23.2	22.0	19.0	19.3	19.3	16.8	18.1	19.6	20.1
Error standard of the mean	0.8	0.8	0.5	0.5	0.6	0.6	0.8	0.2	0.8	0.8	0.5	0.5	0.6	0.6	0.7	0.2	0.2
Percentile 25 th	10.0	10.0	10.0	10.0	10.0	0.0	10.0	10.0	20.0	20.0	20.0	10.0	10.0	10.0	10.0	10.0	10.0
Median	20.0	30.0	20.0	20.0	20.0	10.0	20.0	20.0	30.0	30.0	30.0	30.0	30.0	30.0	20.0	30.0	20.0
Percentile 75th	40.0	40.0	30.0	30.0	30.0	30.0	30.0	30.0	50.0	50.0	40.0	40.0	40.0	40.0	40.0	40.0	40.0
Minimum	0.0	0.0	0.0	0.0	0.0	0.0	0.0	0.0	0.0	0.0	0.0	0.0	0.0	0.0	0.0	0.0	0.0
Maximum	90.0	90.0	90.0	90.0	90.0	100.0	70.0	100.0	100.0	100.0	100.0	100.0	100.0	100.0	100.0	100.0	100.0
Floor effect (%)	9.4	8.9	16.7	17.1	18.7	27.6	22.8	16.6	7.5	6.0	9.1	10.5	11.4	14.7	13.0	10.1	13.3
Ceiling effect (%)	0.0	0.0	0.0	0.0	0.0	0.1	0.0	0.0	0.5	0.1	0.2	0.1	0.4	0.4	0.1	0.2	0.1
																	
**Pain**																	
n=	608	593	1219	1181	987	747	462	5797	679	704	1648	1578	1283	1093	771	7756	13553
Mean	21.5	24.6	24.3	27.0	32.1	28.2	34.0	26.9	28.5	29.2	31.7	32.5	36.5	39.1	40.9	33.5	30.3
Standard deviation	36.9	40.8	31.5	32.6	36.0	28.4	35.0	34.3	33.6	36.2	32.1	31.6	32.1	27.5	30.8	32.0	33.2
Error standard of the mean	1.2	1.3	0.9	0.9	1.0	1.1	1.5	0.4	1.2	1.3	0.8	0.8	0.9	1.1	1.2	0.4	0.3
Percentile 25 th	0.0	0.0	0.0	0.0	0.0	0.0	0.0	0.0	0.0	0.0	0.0	0.0	0.0	0.0	0.0	0.0	0.0
Median	0.0	0.0	0.0	0.0	50.0	0.0	50.0	0.0	0.0	0.0	50.0	50.0	50.0	50.0	50.0	50.0	50.0
Percentile 75th	50.0	50.0	50.0	50.0	50.0	50.0	50.0	50.0	50.0	50.0	50.0	50.0	50.0	50.0	50.0	50.0	50.0
Minimum	0.0	0.0	0.0	0.0	0.0	0.0	0.0	0.0	0.0	0.0	0.0	0.0	0.0	0.0	0.0	0.0	0.0
Maximum	100.0	100.0	100.0	100.0	100.0	100.0	100.0	100.0	100.0	100.0	100.0	100.0	100.0	100.0	100.0	100.0	100.0
Floor effect (%)	63.0	58.9	56.7	53.2	45.6	50.1	41.7	53.5	50.2	52.2	48.3	45.7	39.9	37.6	34.6	44.8	49.1
Ceiling effect (%)	6.0	8.0	5.3	7.1	9.9	6.4	9.7	7.4	7.1	10.6	11.6	10.6	13.0	15.8	16.4	11.8	9.6
																	
**Disability**																	
n=	608	593	1219	1180	986	747	462	5795	680	704	1647	1578	1283	1094	771	7757	13552
Mean	2.4	2.3	1.8	2.7	2.3	1.6	2.1	2.2	2.6	2.8	4.2	3.1	2.8	2.3	1.9	3.0	2.6
Standard deviation	15.0	16.1	11.9	14.7	14.5	10.5	14.0	13.7	14.2	13.5	16.3	14.9	14.4	10.5	10.4	14.0	13.9
Error standard of the mean	0.5	0.5	0.3	0.4	0.4	0.4	0.6	0.2	0.5	0.5	0.4	0.4	0.4	0.4	0.4	0.2	0.1
Percentile 25 th	0.0	0.0	0.0	0.0	0.0	0.0	0.0	0.0	0.0	0.0	0.0	0.0	0.0	0.0	0.0	0.0	0.0
Median	0.0	0.0	0.0	0.0	0.0	0.0	0.0	0.0	0.0	0.0	0.0	0.0	0.0	0.0	0.0	0.0	0.0
Percentile 75th	0.0	0.0	0.0	0.0	0.0	0.0	0.0	0.0	0.0	0.0	0.0	0.0	0.0	0.0	0.0	0.0	0.0
Minimum	0.0	0.0	0.0	0.0	0.0	0.0	0.0	0.0	0.0	0.0	0.0	0.0	0.0	0.0	0.0	0.0	0.0
Maximum	100.0	100.0	100.0	100.0	100.0	100.0	100.0	100.0	100.0	100.0	100.0	100.0	100.0	100.0	100.0	100.0	100.0
Floor effect (%)	95.9	96.4	97.3	96.0	96.8	97.9	97.0	96.7	95.7	94.9	93.7	95.9	96.3	96.8	97.2	95.6	96.1
Ceiling effect (%)	0.7	1.0	0.8	1.4	1.3	1.0	1.2	1.1	0.9	0.5	2.1	2.0	1.9	1.4	0.9	1.6	1.3

* Weighted by number of eligible persons in the household and imputed from 1999 INSEE National Census data.

We found a floor effect for anxiety, perceived health and depression scores (6.4, 11.3 and 12.4%, respectively) and a strong floor effect for pain and disability scores (44.6 and 95.4%, respectively) (Table 3). Ceiling effects were moderate for physical health (11.3%), self-esteem (16%) and mental health (16.2%) and strong for perceived health (53.9%).

## Discussion

The DUKE questionnaire has been used for many years to describe HRQoL in different patient populations but has not been used for a general population. This is the first study presenting norms for the DUKE for French adolescents and adults. These normative data will be useful to researchers who wish to use the DUKE for health assessment and to clinical practitioners in daily practice.

The production of HRQoL community norms is important because they provide expected reference values to evaluate groups or individuals' HRQoL. Norms allow for appreciating the impact of diseases on HRQoL by comparing patients' HRQoL with normative data. However, some authors have suggested that norm-based interpretation in this situation may be irrelevant [35,36] because the impact of the disease could be underestimated. This situation would be the case mainly in longitudinal studies if patients changed their way of estimating HRQoL over time because of their experience with disease or treatment, the response-shift phenomenon. Humans actively construct meaning from their environment and display a range of cognitive mechanisms to continually adapt to changing circumstances. Response shift refers to a change in the meaning of one's evaluation of a construct as a result of a change in one's internal standards of measurement, values or construct definition. Therefore, people might give different answers on patient-reported outcome measures over time, because their HRQoL has changed and because they might have changed their perception on what health or HRQoL means to them [37,38]. However, comparing values between patients and the general population can be problematic with scales that have been developed in a hospital setting, but is not the case for the DUKE.

In public health, the continual monitoring of population HRQoL with validated instruments gives public health agencies data on current health for assessing, protecting, and promoting population health. Tracking population HRQoL over time also helps identify health disparities, evaluate progress on achieving broad health goals, and inform healthy public policy makers. These applications complement those of clinical research and practice, where HRQOL assessment measures patient-reported outcomes from medical, surgical, and behavioural interventions. In epidemiological research, these measures are particularly relevant to the field of chronic disease epidemiology by providing direct evidence of the considerable population burden of long-term health conditions such as disability, arthritis, obesity, asthma or diabetes. As previously mentioned, clinicians and researchers should carefully define their research questions related to patient-reported outcomes before selecting the instrument to use, by structure and content criteria and perhaps according to the availability of normative data.

### Methodological considerations

We found relatively low internal consistency and a strong floor effect with the DUKE. Similar limitations were reported in young people [39] and in dementia [16], and in the French validity study of a cohort of 963 people from the general population, in which the Cronbach α varied from 0.63 to 0.81 [12]. However, this limitation should be moderately weighed because the use of the Cronbach α to assess the psychometric qualities of a HRQoL questionnaire might be inappropriate when the construct validity generates dimensions with few items. The Cronbach α is sensitive to the number of items in the dimension; with increasing number of items, the Cronbach α is likely to increase. In addition, the lower the mean inter-item correlation, the lower the Cronbach α.

We also showed some moderate and high floor effects in dysfunction measures (anxiety, depression, pain and disability) of the DUKE, which indicates poor discrimination properties. This finding was not surprising in a sample from a general population, which is, on average, in good health. These dimension scores are probably sensitive to the impact of disease, as we observed in other studies in patient samples [21,40].

The response rate of the 2005 Health Barometer telephone survey was about 64% (30,514 participants in the 2005 Health Barometer of almost 48,000 contacted), which is lower than the response rate of mail surveys. To be representative of French population, data collected from 2005 Health Barometer have been weighted by number of eligible persons in the household (and by the number of landline phones in the household) and imputed from 1999 INSEE National Census data on gender, age, geographic area and size of agglomeration. In this way, the sample used for this study was representative of these criteria of the French population aged 12 to 75 years old, who speak French and have a landline phone. Characteristics of subjects selected (n = 17,733) and not selected (n = 8,889) for the HRQoL survey group were similar, but despite these precautions and checks, we cannot totally exclude the existence of selection bias.

Finally, the "next-birthday" method used in this national survey [30] to select the person to answer the questions can generate a low "self-selection" phenomenon. However, the results of the selection obtained with this method were very close to those expected. We could have used the Kish method, but it requires, before the selection, describing exactly the whole family, more time and more risk of generating refusals than does the next-birthday method.

## Conclusions

We present HRQoL norms for all dimensions of the DUKE for adolescents and adults in France. These norms could be used as a reference for other studies assessing HRQoL, for specific illnesses, and for international comparisons.

## List of abbreviations

HRQoL: Health-related quality of life; DUKE: DUKE health profile questionnaire; INPES: Institut National de Prévention et d'Education pour la Santé (French National Institute for Prevention and Health Education);

## Competing interests

All the authors declare that they have no competing interests.

## Authors' contributions

All authors read and approved the final manuscript.

Each author has made substantive intellectual contributions to this multicentre study:

CB: statistical analysis, writing manuscript; MLE: statistical analysis, manuscript revision; CPG: manuscript revision, AG: conception of study, manuscript revision, SR: conception of study, manuscript revision; JFC: conception of study, manuscript revision; FG: conception of study, manuscript revision and SB: conception of study manuscript revision and study supervision.

## Pre-publication history

The pre-publication history for this paper can be accessed here:

http://www.biomedcentral.com/1471-2458/11/401/prepub
